# Operative Exposure, Mentorship, and Gender: A Narrative Review of the Determinants of Subspecialty Fellowship Pursuit in Orthopedic Surgery Residents

**DOI:** 10.7759/cureus.110261

**Published:** 2026-06-04

**Authors:** Joseph Salem-Hernández, Derick Rodríguez-Reyes, José Bossolo-Flores, Luis Barreto-Solá, Norman Ramírez

**Affiliations:** 1 Department of Orthopedic Surgery, Ponce Health Sciences University, Ponce, PRI

**Keywords:** fellowship training, gender representation, mentorship, operative exposure, orthopaedic surgery, residency education, subspecialty selection, workforce diversity

## Abstract

Fellowship training has become a near-universal component of orthopedic surgical education, yet the factors driving residents toward specific subspecialties remain incompletely characterized. Identifying these determinants is essential for residency program directors, fellowship training programs, and professional societies seeking to optimize workforce development and address persistent disparities in subspecialty representation. A comprehensive narrative review of 75 peer-reviewed studies was conducted using systematic searches of PubMed, Google Scholar, and SciSpace. Quantitative survey data, national fellowship match statistics, and qualitative findings were synthesized to characterize factors influencing fellowship selection across five major orthopedic subspecialties: sports medicine, spine surgery, arthroplasty (adult reconstruction), pediatric orthopedics, and trauma. Fellowship selection is a multifaceted decision driven by intrinsic motivation, operative exposure in residency, mentorship relationships, and practical considerations that vary substantially across subspecialties. Sports medicine applicants prioritize clinical outcomes, personal interest, and operative variety. Arthroplasty candidates emphasize intellectual challenge and mentorship quality, whereas pursuit of a spine fellowship shows the strongest correlation with operative volume during residency (108.4 ± 50.7 vs. 74.4 ± 60.2 cases, p < 0.01). Pediatric orthopedics attracts the highest proportion of female applicants at 25% and is distinguished by program directors' emphasis on interview performance and letters of recommendation. Significant gender disparities persist, with female representation ranging from 3% in spine to 25% in pediatrics against an overall mean of 11%. Quantitative trauma-specific selection data remain limited in the published literature. Personal interest and subspecialty passion are universal drivers in fellowship selection, while operative volume, mentorship quality, intellectual challenge, and financial considerations shape distinctive applicant profiles across subspecialties. The more-than-eightfold variation in female representation across fellowships suggests the need for targeted mentorship initiatives, culture assessments, and increased visibility of role models, particularly in spine surgery.

## Introduction and background

The decision to pursue fellowship training represents a critical juncture in the career trajectory of orthopedic surgery residents. With increasing subspecialization across the field, understanding the factors that influence fellowship choices has become essential for residency program directors, training programs, and professional societies seeking to optimize workforce development and address disparities in subspecialty representation [1].

This review examines the factors influencing orthopedic surgery residents' decisions to pursue fellowship training in five major subspecialties: sports medicine, spine surgery, arthroplasty (adult reconstruction), pediatric orthopedics, and trauma. Drawing on a comprehensive narrative review of 75 studies identified through systematic searches of PubMed, Google Scholar, and SciSpace, this analysis synthesizes quantitative survey data, national match statistics, and qualitative insights to characterize the decision-making landscape for each subspecialty [2,3]. Note that while five subspecialties serve as the primary focus of the narrative analysis, the summary figures include comparative data across eight fellowship subspecialties derived from the reviewed literature; this distinction is maintained throughout the manuscript.

**Figure 1:**
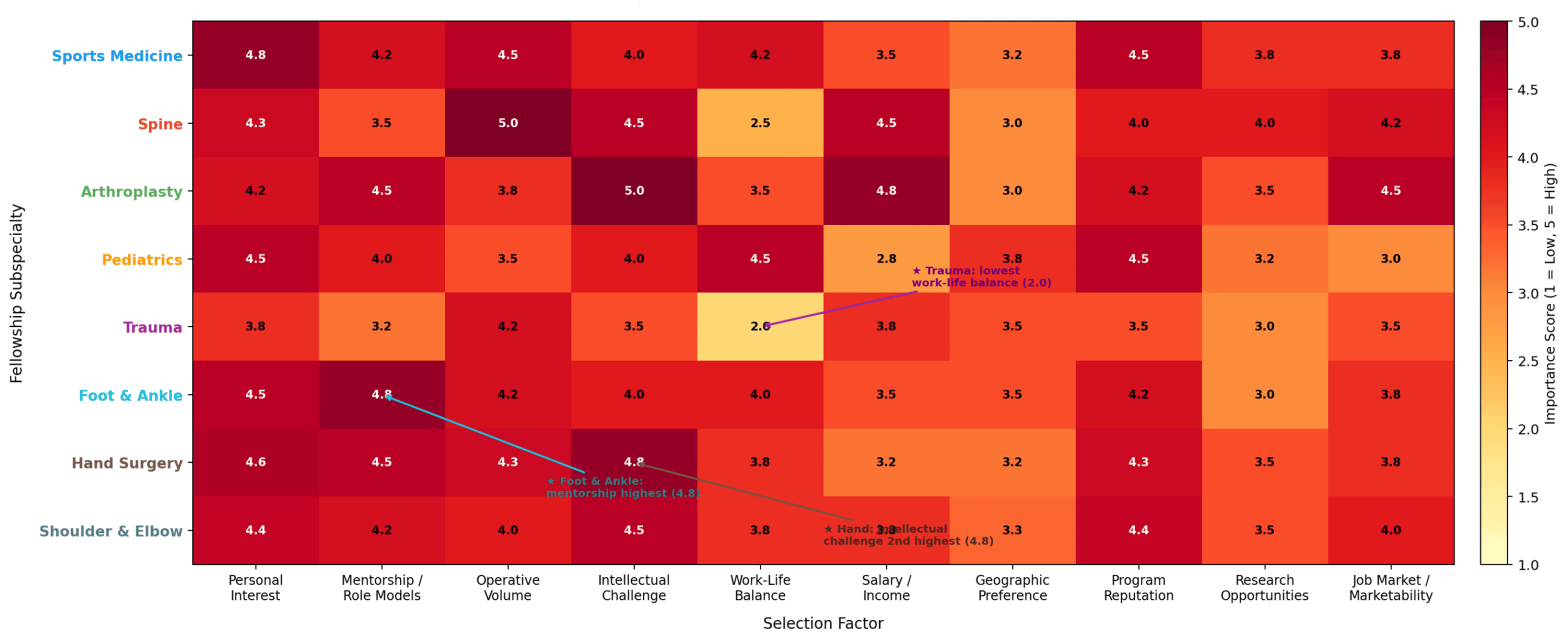
Importance of fellowship selection factors by orthopedic subspecialty Heatmap displaying the relative importance of ten fellowship selection factors across eight orthopedic subspecialties on a literature-derived scale of 1 (low importance) to 5 (high importance). Sports Medicine shows particularly high scores for personal interest (4.8) and program reputation (4.5). Spine and Arthroplasty lead in intellectual challenge (5.0). Trauma demonstrates the lowest work-life balance priority (2.0). Data derived from Butler et al. [2], n = 253; Daniels et al. [3], n = 1,823; Haddad et al. [6], multi-year national ACGME cohort; Enata et al. [7], n = 107; and Baweja et al. [8], n = 62. Created using GraphPad Prism (GraphPad Software, LLC., Boston, MA, USA).

The factors examined include personal interest and disease passion, mentorship and role models, operative volume and exposure during residency, intellectual challenge, work-life balance and lifestyle considerations, salary and income potential, geographic preferences, program reputation and faculty, research opportunities, and job market considerations. By comparing these factors across subspecialties, this review identifies both common themes and subspecialty-specific patterns that shape fellowship selection decisions. Figure 1 provides an overview of the relative importance of all ten selection factors across eight orthopedic subspecialties [4,5].

Understanding these determinants has practical implications for multiple stakeholders. Residency programs can design curricula and mentorship initiatives that provide balanced exposure to subspecialties. Fellowship programs can tailor recruitment strategies to highlight factors most valued by their target applicant pool. Professional societies can develop interventions to address gender disparities and promote diversity in underrepresented subspecialties [6-8].

## Review

Materials and methods

Systematic literature searches were conducted in PubMed, Google Scholar, and SciSpace using the following search terms: "orthopaedic fellowship selection", "fellowship training determinants", "subspecialty choice orthopaedic surgery", "gender orthopaedic fellowship", "mentorship orthopaedic residency", and related MeSH terms. Searches were conducted through December 2024 without language restrictions. Included studies were peer-reviewed publications reporting quantitative survey data, national fellowship match statistics, or qualitative findings related to fellowship selection factors in orthopedic surgery. Studies were excluded if they focused exclusively on non-orthopedic surgical specialties or reported no data relevant to fellowship selection decisions.

Two authors (J.S.-H. and D.R.-R.) independently screened titles and abstracts, and all five co-authors conducted full-text reviews. Disagreements were resolved by consensus discussion. Because this is a narrative review, no formal risk-of-bias tool was applied; however, the methodological limitations of the included studies, primarily survey-based designs subject to selection bias, non-response bias, and heterogeneity in questionnaire design, target populations, response rates, and outcome definitions, are acknowledged explicitly in the Limitations section. Study conclusions are discussed throughout the manuscript in the context of their sample size and methodological design. Studies were not formally weighted by sample size; where quantitative comparisons are presented in figures, the contributing sample sizes are noted in the figure legends. Key study sample sizes are as follows: Butler et al. [2], n = 253 orthopedic residents; Daniels et al. [3], n = 1,823 orthopedic residents; Haddad et al. [6], multi-year national ACGME cohort; Enata et al. [7], n = 107 women orthopedic surgeons; and Baweja et al. [8], n = 62 sports medicine fellowship applicants.

Sports medicine fellowship selection factors

Sports medicine is among the most popular fellowship choices for orthopedic surgery residents, driven by both intrinsic and extrinsic motivational factors. Survey data reveal that sports medicine applicants place exceptionally high value on clinical outcomes, personal interest, and the quality of their residency exposure to the subspecialty.

Primary Motivating Factors

Quantitative surveys of sports medicine fellowship applicants and trainees (n = 253) demonstrate that disease prognosis and clinical outcomes rank as the highest motivating factor, with 90% of residents selecting sports medicine as important [2]. Personal interest follows closely at 87%, while residency experience and exposure rank third at 83%. Additional highly rated factors include specific surgical skills (81%), disease pathology and patient population characteristics (77%), expected workload and lifestyle considerations (73%), the influence of role models and mentors (70%), and patient volume and variety (67%) [2]. These survey findings are consistent with the interpretation that sports medicine attracts residents who are intrinsically motivated by the nature of the patient population; however, this characterization is the authors' synthesis of the available data and should be understood as a proposed explanatory framework rather than a directly measured finding.

Program Selection Priorities

When evaluating specific fellowship programs, sports medicine applicants prioritize different factors than those driving their initial subspecialty choice. A ranking study of program attributes found that variety and complexity of surgical exposure ranked as the most important program factor (mean rank 2.16), followed by autonomy in surgical decision-making (3.72) and faculty reputation (4.05). Geographic location ranked lower in importance (5.68), suggesting that applicants are willing to relocate for high-quality training experiences [8]. The emphasis on surgical variety and autonomy reflects sports medicine's broad scope, encompassing arthroscopic procedures, ligament reconstruction, cartilage restoration, and fracture management across multiple joints.

Role of Faculty and Program Reputation

Multiple studies confirm that sports medicine applicants consistently place high value on faculty members and overall program reputation when making rank decisions [1,4]. The quality and national recognition of fellowship faculty serve as key differentiators among programs, likely reflecting applicants' desire to train with leaders in the field and to establish professional networks that will support their future careers.

Residency Operative Experience

Longitudinal analysis of residency case logs provides compelling evidence that operative exposure during residency influences subspecialty selection. Residents who ultimately chose sports medicine fellowships logged significantly more sports-related cases during residency (278.5 ± 105.8 cases) compared to colleagues who selected other subspecialties (229.0 ± 93.9 cases, p < 0.01) [3]. This correlation suggests that early and substantial exposure to sports medicine procedures during residency reinforces interest and competence, ultimately channeling residents toward sports medicine fellowship training.

Arthroplasty and adult reconstruction fellowship selection factors

Arthroplasty (adult reconstruction) fellowship selection is characterized by distinct motivational patterns that emphasize intellectual engagement and mentorship relationships. While sharing some common factors with other subspecialties, arthroplasty attracts residents through a unique combination of technical challenge, predictable outcomes, and the opportunity to restore function in an aging population.

Intellectual Challenge and Mentorship

**Figure 2:**
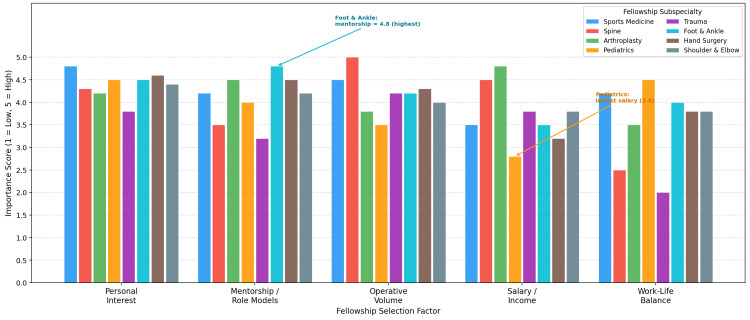
Top five fellowship selection factors across orthopedic subspecialties Grouped bar chart comparing the importance of five key selection factors across subspecialties on a scale of 1-5. Arthroplasty shows the highest importance scores for intellectual challenge (5.0) and salary/income potential (4.8). Sports medicine leads in personal interest (4.8). Spine demonstrates the highest operative volume importance score (5.0). Foot and ankle shows the highest mentorship score (4.8). Data derived from Butler et al. [2], n = 253, and Daniels et al. [3], n = 1,823. Created using GraphPad Prism (GraphPad Software, LLC., Boston, MA, USA).

Survey research identifies intellectual challenge and the presence of role models and mentors as the highest-priority factors among residents pursuing arthroplasty fellowships [2]. This emphasis on intellectual stimulation distinguishes arthroplasty from subspecialties that are more heavily driven by lifestyle considerations or patient population preferences. The technical complexity of primary and revision joint replacement, the evolving landscape of implant technology, and the problem-solving required for complex reconstructions appeal to residents who value cognitive engagement in their surgical practice. A comparison of the top five selection factors across subspecialties is presented in Figure 2. Based on the reviewed evidence that intellectual challenge and mentorship quality are the most highly rated selection factors among arthroplasty applicants, a potential implication is that programs may benefit from prioritizing early mentorship opportunities and highlighting the intellectual challenges inherent in joint reconstruction, though this recommendation requires prospective validation.

Subspecialty Popularity and Residency Operative Exposure

In a multicenter survey of orthopedic residents, arthroplasty was reported as the presumptive subspecialty choice by 17.3% of respondents, indicating moderate popularity relative to other subspecialties [2]. Unlike sports medicine and spine, arthroplasty did not demonstrate a statistically significant association between residency operative volume and fellowship selection. Residents who chose arthroplasty fellowships did not log significantly more arthroplasty cases during residency compared to peers who selected other subspecialties (p = 0.18) [3]. This finding suggests that intellectual appeal, mentorship quality, and perceived career opportunities may be more influential than operative volume in driving arthroplasty fellowship selection.

Implications for Career Messaging

Highlighting intellectual challenges, technological innovation, and opportunities for early mentorship in adult reconstruction may effectively increase resident interest in arthroplasty fellowships [2]. Residency programs should ensure that arthroplasty rotations emphasize problem-solving, complex case management, and exposure to revision surgery to appeal to intellectually motivated residents.

Spine fellowship selection factors

Spine surgery fellowship selection exhibits the strongest correlation with residency operative exposure among all orthopedic subspecialties examined. This subspecialty also demonstrates notable gender disparities and attracts residents who prioritize technical complexity and high operative volume.

Operative Volume and Fellowship Selection

**Figure 3:**
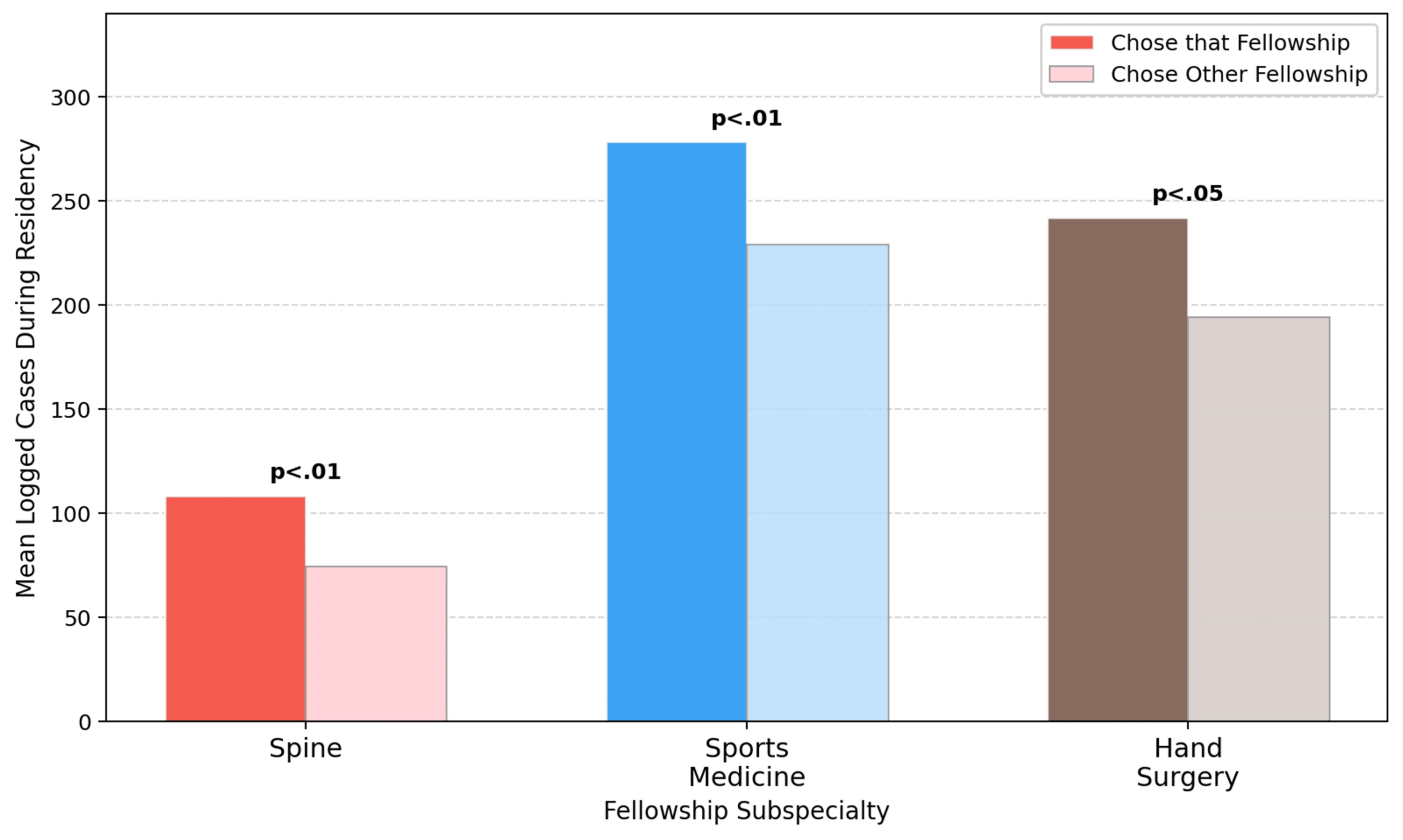
Residency operative volume by fellowship choice across orthopedic subspecialties Grouped bar chart comparing mean logged operative cases during residency for residents who chose versus did not choose each subspecialty fellowship. Spine fellows logged significantly more spine cases than non-spine-bound peers (108.4 ± 50.7 vs. 74.4 ± 60.2 cases, p < 0.01), and Sports Medicine fellows logged significantly more sports-related cases than peers selecting other subspecialties (278.5 ± 105.8 vs. 229.0 ± 93.9 cases, p < 0.01). Arthroplasty did not demonstrate a statistically significant volume-selection association (p = 0.18). Data derived from Daniels et al. [3], n = 1,823. Created using GraphPad Prism (GraphPad Software, LLC., Boston, MA, USA).

Longitudinal analysis of residency case logs reveals a robust association between spine operative experience during residency and subsequent selection of spine fellowship. Residents who chose spine fellowships logged significantly more spine cases during residency (108.4 ± 50.7 cases) compared to peers who selected other subspecialties (74.4 ± 60.2 cases, p < 0.01) [3]. This represents the strongest volume-selection correlation among all subspecialties studied, suggesting that operative exposure plays a particularly influential role in channeling residents toward spine surgery. The mechanism likely involves increased technical confidence, mentorship with spine faculty, and firsthand experience of the intellectual and technical demands of spinal reconstruction. A direct comparison of operative volume by fellowship choice across subspecialties is shown in Figure 3.

Intellectual Challenge and Technical Complexity

Spine surgery ranks among the most intellectually challenging subspecialties (score 5.0 on a 5-point scale), reflecting the anatomical complexity, neurological considerations, and technical demands of spinal reconstruction [2]. This intellectual appeal attracts residents seeking cognitively demanding surgical challenges and comfortable with the high-stakes nature of spine surgery (Figures 1-2).

Trainee Priorities Across Training Levels

Research indicates that residents' priorities evolve over the course of training, with implications for spine fellowship selection. Senior trainees (PGY-4 and PGY-5) place greater emphasis on case variety and intellectual stimulation, while junior trainees place greater weight on lifestyle factors, on-call responsibilities, geographic location, and financial compensation [2]. Programs should provide early exposure to spine surgery while addressing lifestyle and compensation questions transparently to maintain resident interest throughout training.

Gender Representation

Spine surgery exhibits the most severe gender disparity among all orthopedic fellowships, with only 3% female representation in a multi-year national match analysis [6]. This marked disparity, discussed in detail in the Gender Representation section below, suggests that gendered faculty composition, mentorship patterns, or perceived cultural factors may influence female residents' subspecialty choices and demand targeted institutional and professional society intervention.

Pediatric orthopedics fellowship selection factors

Pediatric orthopedics represents a distinctive fellowship pathway characterized by unique selection criteria, a high proportion of women, and program director priorities that differ substantially from those of other subspecialties.

Program Director Selection Priorities

Research examining pediatric orthopedic fellowship program directors' perspectives reveals that interview performance and letters of recommendation rank as the most important selection criteria for ranking applicants. Additional factors rated as important include the reputation of the applicant's residency program, personal connections and networking, and life experiences demonstrating commitment to pediatric care [5]. This emphasis on holistic evaluation and interpersonal factors distinguishes pediatric fellowship selection from subspecialties that may place greater weight on research productivity or technical skills.

Motivations and Gender Representation

**Figure 4:**
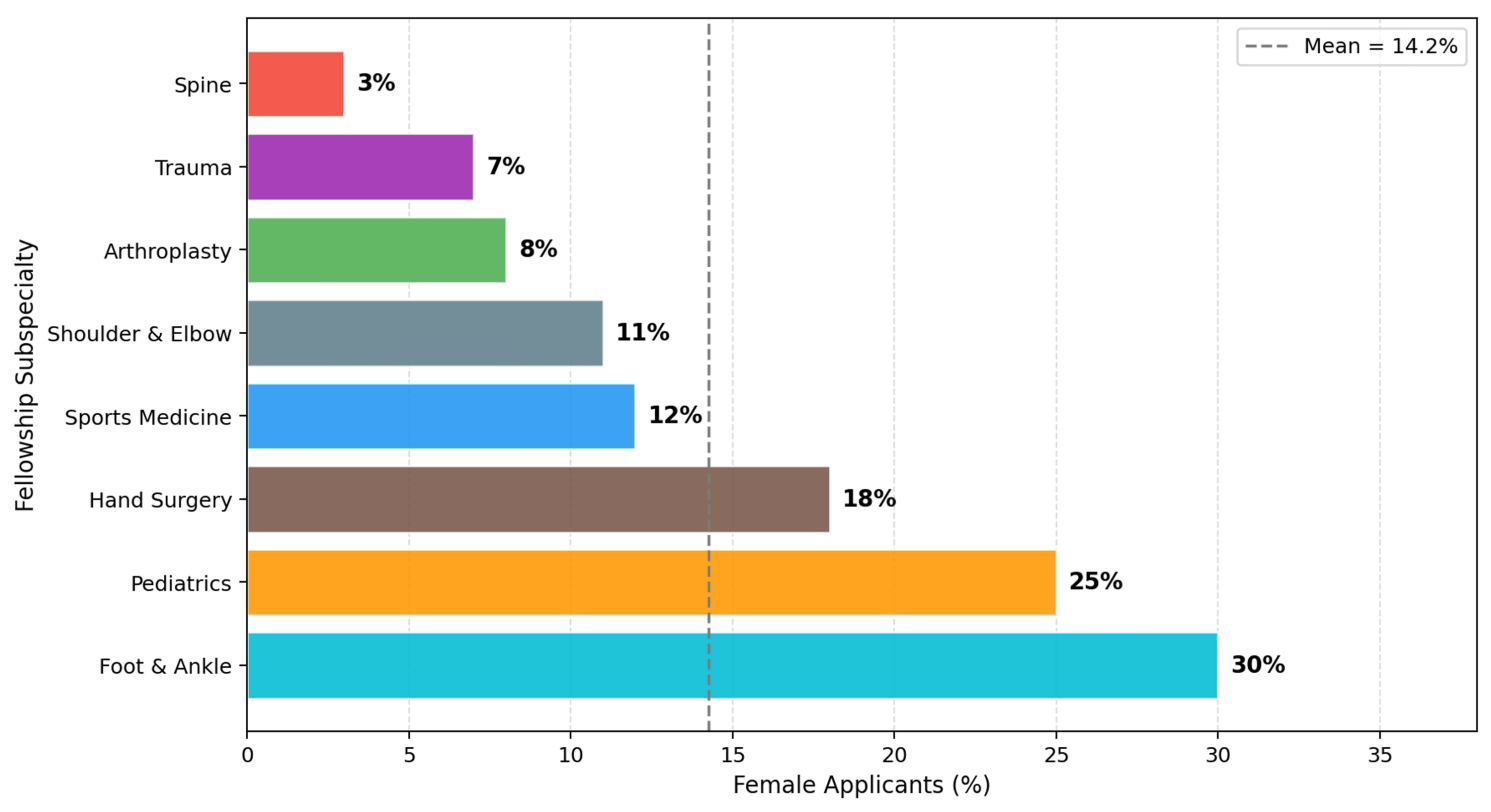
Proportion of female applicants by orthopedic fellowship subspecialty Horizontal bar chart displaying the percentage of female applicants across eight orthopedic fellowship subspecialties. Female representation ranges from 3% in spine surgery to 30% in foot and ankle, with an overall mean of 14.2%. Pediatric orthopedics attracts 25% female applicants, substantially exceeding the mean. Data derived from Haddad et al. [6], multi-year national ACGME cohort. Created using GraphPad Prism (GraphPad Software, LLC., Boston, MA, USA).

Pediatric applicants place relatively lower emphasis on salary and income potential (score 2.8, the lowest among all subspecialties examined) and demonstrate the highest proportion of female fellowship applicants among all orthopedic subspecialties, at 25% in national match data (Figure 4) [6]. This substantially exceeds the overall mean of 11% across all orthopedic fellowships and represents more than eight times the female representation in spine surgery. Pediatric orthopedics is often perceived as offering more controllable schedules and better work-life integration, and the presence of more female faculty may provide visible role models that attract women residents (Figures 1-2).

Women Surgeons' Fellowship Decisions

Survey data from Enata et al. (n = 107) indicate that 79% of women orthopedic surgeons reported that the presence or absence of female role models did not consciously influence their fellowship choice [7]. This finding appears to be at odds with the hypothesis that role model scarcity drives gender disparities at the population level; however, self-reported surveys may not fully capture unconscious or structural influences, and population-level gender distributions suggest that unmeasured factors continue to shape subspecialty representation regardless of individual-level perceptions.

Trauma fellowship selection factors

Trauma orthopedics represents a critical subspecialty focused on acute injury management, polytrauma care, and complex fracture reconstruction. However, the available literature provides limited quantitative data on trauma-specific fellowship-selection factors compared with other subspecialties, representing a significant gap that future research must address.

Available Evidence and Inferred Priorities

Based on the limited available evidence and comparative data across subspecialties, trauma fellowship applicants appear to place moderate emphasis on personal interest, mentorship, and operative volume [2,3]. Trauma demonstrates a notably lower priority for work-life balance considerations compared to other subspecialties (score 2.0, the lowest among those examined), suggesting that trauma applicants may self-select for tolerance of demanding call schedules and unpredictable hours. Female representation in trauma fellowship programs is 7%, below the overall mean of 11% but substantially higher than the 3% in spine surgery [6].

Need for Future Research

Future research should employ rigorous survey methodologies, comparable to those used in other subspecialties, to characterize trauma applicants' motivations, program selection priorities, and the influence of residency experiences on the pursuit of trauma fellowships. Understanding these factors would support more effective recruitment, mentorship, and workforce planning for this essential subspecialty.

Trends and anomalies across subspecialties

**Figure 5:**
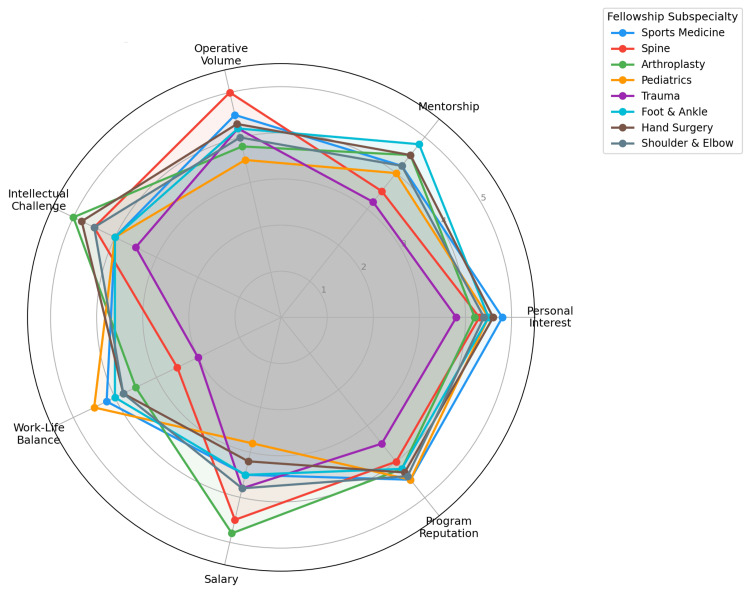
Multi-factor fellowship selection profile by orthopedic subspecialty Radar chart comparing seven key selection factors across eight orthopedic fellowship subspecialties. Distance from the center indicates importance on a scale of 1-5. Spine and arthroplasty demonstrate high operative volume and the importance of intellectual challenge. Pediatrics shows high scores in personal interest and work-life balance. Trauma displays the lowest work-life balance priority. Data derived from Butler et al. [2], n = 253; Daniels et al. [3], n = 1,823; Haddad et al. [6], multi-year national ACGME cohort; Enata et al. [7], n = 107; and Baweja et al. [8], n = 62. Created using GraphPad Prism (GraphPad Software, LLC., Boston, MA, USA).

Comparative analysis across the five orthopedic fellowship subspecialties reveals both common themes and striking anomalies that illuminate the complex landscape of fellowship selection decisions. The multi-factor selection profiles across all subspecialties are summarized in Figure 5.

Spine Surgery as a Critical Anomaly

Spine surgery's 3% female representation is the most severe gender disparity among all orthopedic subspecialties examined, representing less than one-third of the already low overall mean [6]. Potential contributing factors include perceived lifestyle demands, a relative scarcity of female spine surgery faculty, and subspecialty culture regarding gender inclusivity. It should be noted that the physical demands of spine surgery are not uniquely greater than those of other orthopedic subspecialties; arthroplasty and trauma may involve comparable or greater physical workloads depending on practice patterns and case complexity, and physical demands should therefore not be cited as a spine-specific contributor without supporting evidence. Regarding role models: while it may be hypothesized that the scarcity of female spine faculty limits mentorship visibility and contributes to underrepresentation, survey data from Enata et al. [7] indicate that 79% of women orthopedic surgeons reported role model availability did not consciously influence their subspecialty choice (see Women Surgeons' Fellowship Decisions section for a full discussion). These findings are not contradictory but complementary: individual-level self-report and population-level structural patterns can diverge, and unmeasured or unconscious influences may operate even where residents do not consciously perceive them.

Ionizing Radiation Exposure as an Unaddressed Factor

Notably, none of the 75 studies included in this review examined occupational exposure to ionizing radiation as a factor in fellowship selection, despite its substantial variation across subspecialties. Spine trauma and, depending on practice patterns, adult reconstruction involve regular fluoroscopic exposure. Given the broader literature on occupational radiation risk, malignancy concerns, and considerations for surgeons of reproductive age, this omission in the fellowship selection literature is significant. Future research should investigate whether radiation concerns influence residents' decisions, particularly in spine and trauma fellowships.

Age as an Omitted Variable

Age at the time of residency or fellowship decision-making was not systematically reported in the included studies. Age may influence career priorities, family planning considerations, financial responsibilities, and tolerance for longer or more demanding training pathways. This is particularly relevant when considering international applicability, as in some countries medical careers begin later due to mandatory military service or longer training systems. The absence of age-related data in the fellowship selection literature is noted as a limitation and an area for future research.

Implications for Diversity Initiatives

The substantial gender disparities across orthopedic subspecialties have direct implications for diversity, equity, and inclusion efforts. Subspecialties with low female representation, particularly spine, should develop targeted mentorship programs connecting women residents with faculty committed to supporting women's career development. Programs should assess subspecialty culture and implement interventions to address identified barriers while increasing the visibility of successful women through conference presentations, leadership roles, and residency education [6,7]. Providing accurate information about work-life balance and practice patterns can help dispel misconceptions that may disproportionately deter women from certain subspecialties.

Study limitations

This narrative review has several limitations. First, the reliance on self-reported survey data introduces response and recall biases, as residents may not accurately characterize the factors that influenced their fellowship decisions. Second, the studies included vary considerably in sample size, methodology, and year of publication, limiting direct comparisons across subspecialties. Because this review relies largely on survey-based studies, the potential for selection bias, non-response bias, sampling bias, and heterogeneity in questionnaire design should be explicitly acknowledged; without a formal risk-of-bias assessment, the strength of the conclusions must be interpreted with appropriate caution. Third, the review is restricted to five orthopedic subspecialties, and findings may not generalize to hand surgery, foot and ankle, shoulder and elbow, or musculoskeletal oncology fellowships. Fourth, quantitative trauma-specific fellowship selection data remain sparse in the published literature, precluding robust conclusions for that subspecialty. Fifth, gender is examined as a binary variable across the included studies, which does not capture the full spectrum of gender identity among orthopedic trainees. Sixth, none of the included studies examined occupational exposure to ionizing radiation as a fellowship selection factor, representing a notable gap given its substantial variation across subspecialties. Seventh, age at fellowship decision-making was not systematically reported in the included studies, limiting the ability to assess its interaction with gender, family planning, and other career considerations. Eighth, because most source studies were conducted in the United States, the findings may have limited applicability to orthopedic training systems in other countries; in settings where military service is mandatory, medical careers begin later, or training durations differ, residents may approach fellowship decisions with different financial, family, and career-stage considerations that interact with gender in ways not captured by the current literature. Finally, this review does not capture the perspectives of residents who choose not to pursue fellowship training or who move away from operative subspecialty practice toward community-based or non-operative orthopedic careers; understanding this group may provide important insight into broader career decision-making among orthopedic trainees.

## Conclusions

This narrative review demonstrates that orthopedic fellowship selection is a complex, multifaceted decision that varies substantially across subspecialties. Personal interest and passion for the patient population emerge as universal drivers. At the same time, operative volume, mentorship quality, intellectual challenge, lifestyle considerations, and financial priorities create distinctive applicant profiles for each fellowship pathway. Residency operative exposure plays a critical yet variable role: spine and sports medicine demonstrate strong, statistically significant volume-selection correlations. At the same time, arthroplasty shows no significant association, underscoring that case volume alone does not determine career trajectories. The dramatic divergence in salary prioritization between arthroplasty and pediatrics carries meaningful implications for compensation equity and long-term workforce sustainability in mission-driven subspecialties. The more than eightfold variation in female representation across fellowships, ranging from 3% in spine to 25% in pediatrics, represents the most urgent workforce diversity challenge identified in this review and suggests the need for targeted interventions through mentorship programs, culture assessments, and increased role model visibility. The limited evidence base for trauma fellowship selection factors represents a significant gap requiring future investigation using rigorous, subspecialty-specific survey methodologies. Collectively, these findings provide an evidence-based foundation for optimizing residency curricula, fellowship recruitment strategies, and professional society interventions to support informed career decision-making and a more diverse and equitable orthopedic surgical workforce.
